# Research on Protection of a Three-Level Converter-Based Flexible DC Traction Substation System

**DOI:** 10.3390/s26041350

**Published:** 2026-02-20

**Authors:** Peng Chen, Qiang Fu, Chunjie Wang, Yaning Zhu

**Affiliations:** 1 School of Electrical Engineering and Automation, Tianjin University of Technology, Tianjin 300382, China; chenpeng@email.tjut.edu.cn (P.C.);; 2 Tianjin Key Laboratory of New Energy Power Conversion, Transmission and Intelligent Control, Tianjin 300382, China

**Keywords:** flexible DC traction power supply, three-level converter, ANPC, DC-side short-circuit fault, protection strategy, urban rail transit

## Abstract

With the expansion of urban rail transit, increased train operation density, and the large-scale grid integration of renewable energy such as offshore photovoltaic power, traction power supply systems face stricter requirements for operational safety, power supply reliability and energy utilization efficiency. Offshore photovoltaic power, integrated into the traction power supply network via flexible DC transmission technology, promotes renewable energy consumption, but its random and volatile output overlaps with time-varying traction loads, increasing the complexity of DC-side fault characteristics and protection control. Flexible DC technology is a core direction for next-generation traction substations, and three-level converters (key energy conversion units) have advantages over traditional two-level topologies. However, their P-O-N three-terminal DC-side topology introduces new faults (e.g., PO/ON bipolar short circuits, O-point-to-ground faults), making traditional protection strategies ineffective. In addition, wide system current fluctuation (0.5–3 kA) and offshore photovoltaic power fluctuation easily cause fixed-threshold protection maloperation, and the coupling mechanism among modulation strategies, DC bus capacitor voltage dynamics and fault current paths is unclear. To solve these bottlenecks, this paper establishes a simulation model of the system based on the PSCAD/EMTDC(A professional simulation software for electromagnetic transient analysis in power systems V4.5.3) platform, analyzes the transient electrical characteristics of three-level converters under traction and braking conditions for typical faults, clarifies the coupling mechanism, proposes a condition-adaptive fault identification strategy, and designs a reconfigurable fault energy handling system with bypass thyristors and adaptive crowbar circuits. Simulation and hardware-in-the-loop (HIL) experiments show that the proposed scheme completes fault identification and protection within 2–3 ms, suppresses fault peak current by more than 70%, limits DC bus overvoltage within ±10% of the rated voltage, and has good post-fault recovery performance. It provides a reliable and engineering-feasible protection solution for related systems and technical references for similar flexible DC system protection design.

## 1. Introduction

Urban rail transit has become a cornerstone of modern urban transportation systems due to its advantages, such as a large transportation capacity, high operating speed, and environmentally friendly characteristics. As a key infrastructure for alleviating traffic congestion and improving commuting efficiency, its rapid expansion has led to increasingly stringent requirements for operational safety, power supply reliability, and energy efficiency of traction power supply systems [1,2]. As the “power heart” of urban rail transit, the traction power supply system is responsible for energy transmission, conversion, and fault protection, and its reliability directly determines the stability and efficiency of train operation.

At present, most DC traction power supply systems adopt the architecture of a diode rectifier combined with an independent regenerative energy feedback device. Although this configuration enables the recovery of braking energy, it requires additional equipment such as DC switchgear and feedback transformers, leading to a 30–40% increase in system complexity, a 25–35% rise in construction cost, and a 15–20% reduction in overall reliability [3,4]. These limitations make it difficult for conventional traction power supply systems to meet the growing demands for flexibility and energy efficiency under high-density train operation conditions (e.g., train headway <90 s in megacities) [3,4].

Flexible DC traction power supply technology has therefore emerged as a promising solution for next-generation urban rail transit systems. Owing to its high voltage regulation accuracy (±2%), smooth bidirectional power flow capability, and low harmonic distortion (<3%), flexible DC technology enables the integrated realization of traction power supply and regenerative energy feedback [1]. By replacing traditional rectifier–feedback architectures, it significantly simplifies system structure and improves energy utilization efficiency by 15–20%, making it a key development direction for modern traction substations.

Among various flexible DC traction power supply solutions, the three-level converter based on the Active Neutral Point Clamped (ANPC) topology has attracted increasing attention in recent years. By employing fully controlled power devices, the ANPC three-level converter enables the integrated realization of rectification and inversion functions, effectively addressing the issues of energy back feeding, equipment redundancy, and poor power quality associated with conventional traction power supply architectures. In addition, the voltage stress of each switching device is reduced to half of that in two-level converters, while the output voltage waveform exhibits lower harmonic content. These features provide significant engineering advantages for high-capacity traction power supply systems, and pilot applications have already been reported in several urban rail transit projects [2,5,6].

Despite these advantages, the introduction of the P–O–N three-terminal DC-side topology fundamentally alters the fault characteristics and transient behaviors of traction power supply systems, creating critical research gaps that remain unaddressed:

New Fault Modes: Compared with traditional two-level systems (mainly PN bipolar short circuits and pole-to-ground faults), three-level systems introduce additional fault types, including PO and ON bipolar short circuits as well as neutral-point-related faults. These newly introduced fault modes are characterized by extremely high current rise rates (up to 10 kA/ms, 3–5 times higher than two-level systems) and fault current paths that are essentially different from those of conventional short circuits [7,8]. The existing literature primarily focuses on PN faults, with limited systematic analysis of PO/ON faults under actual traction/braking conditions. For MMC-HVDC systems, similar challenges in fast fault identification have been addressed by novel current-limiting DC circuit breaker-based schemes, which achieve rapid fault isolation through boundary protection and traveling wave blocking [9], but such approaches have not been adapted to the unique P-O-N topology and traction/braking operating conditions of three-level converter-based traction systems.

Fixed-Threshold Protection Limitations: During traction and braking modes, system currents typically fluctuate within a wide range of 0.5–3 kA, which makes conventional fixed-threshold-based protection schemes prone to maloperation (misjudgment rate >15%) due to operating condition confusion. Moreover, the selection of zero vectors in Space Vector Pulse Width Modulation (SVPWM) strategies significantly influences fault current transient characteristics, but the quantitative impact of modulation strategies on protection criteria has not been sufficiently clarified [10,11]. For true bipolar MMC-HVDC systems, research has shown that accurate fault current calculation is critical for optimizing protection thresholds, as transient characteristics are closely coupled with system topology and modulation patterns [12], providing a reference for addressing similar issues in traction system protection design.

Inadequate Fault Energy Handling: Conventional fault energy handling approaches (e.g., fast fuses, traditional crowbar circuits) are designed for simple current paths and single-capacitor structures. These methods are inadequate for three-level systems with multi-branch capacitor coupling and superimposed energy feedback paths, often resulting in capacitor overvoltage (exceeding rated voltage by 30–50%), device thermal stress (diode current exceeding rated value by 200–300%), or secondary fault impacts [13,14,15].

Moreover, many protection strategies reported in the literature are developed based on fixed threshold settings. Under practical traction and braking conditions, where operating currents vary significantly, such strategies are prone to misjudgment and delayed response. In addition, conventional fault energy handling approaches, such as fast fuses and traditional crowbar circuits, are generally designed for simple current paths and single-capacitor structures. These methods are often inadequate for three-level systems with multi-branch capacitor coupling and superimposed energy feedback paths, and may result in capacitor overvoltage, device thermal stress, or secondary fault impacts [10,11,12].

To address the above gaps, this paper sets the following primary research objectives:

To model and systematically analyze the transient characteristics of PN/PO/ON bipolar short-circuit faults in three-level ANPC converters under both traction and braking conditions, clarifying the coupling mechanism among modulation strategies, DC bus capacitor voltage dynamics, and fault current paths.

To develop a condition-adaptive fault identification strategy that integrates real-time operating mode recognition (traction/braking) with fault characteristic extraction, overcoming the limitations of fixed-threshold methods.

To design a reconfigurable energy handling system (bypass thyristor + adaptive crowbar) that provides differentiated protection for different fault types, effectively managing both overcurrent and overvoltage risks.

To validate the proposed scheme through PSCAD simulations and hardware-in-the-loop (HIL) experiments, quantifying its performance advantages over conventional methods.

This paper takes the 1500 VDC traction power supply system of a metro project in Tianjin as the engineering background. The research results can provide theoretical support and technical reference for the engineering application of flexible DC traction power supply systems, and have important practical significance for ensuring the operational safety of rail transit.

This paper focuses on the DC-side fault protection of the flexible DC traction system based on the three-level ANPC converter. The introduction points out the limitations of traditional traction power supply systems and the advantages of flexible DC technology, analyzes the characteristics of typical DC-side short-circuit faults (including positive-negative pole, positive pole-to-ground and negative pole-to-ground short circuits) and the shortcomings of conventional protection strategies, and clarifies the research objectives of this paper. Section 2, the system modeling section, establishes a 1500 V DC traction system simulation model based on the PSCAD/EMTDC simulation platform, and defines the relevant topological structures and parameter configurations. Section 3, the fault analysis section, systematically investigates the fault characteristics of the three types of DC-side short-circuit faults under traction and braking operating conditions. Section 4, the protection scheme design section, proposes an operating condition-adaptive fault identification strategy and constructs an integrated protection system with differentiated protection functions. Section 5, the experimental verification section, verifies the proposed protection scheme through PSCAD simulations and hardware-in-the-loop (HIL) tests; the conclusion section summarizes the advantages and engineering application value of the proposed scheme, and considers future research directions [16,17,18,19,20].

## 2. System Structure and Simulation Modeling of the DC Traction Power Supply System

### 2.1. Structure of the Traction Power Supply System

**Figure 1:**
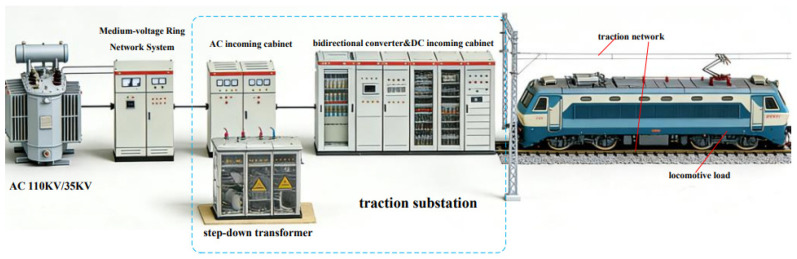
Overall configuration of the DC traction power supply system.

The urban rail transit traction power supply system is responsible for supplying and transmitting electrical energy required for train operation, auxiliary equipment, and related facilities. It mainly consists of a medium-voltage ring network, traction substations, and the traction power supply network. The overall electrical structure of the traction power supply system is illustrated in Figure 1 [21].

In modern urban rail transit systems, electric traction power is generally supplied from the public utility grid. High-voltage three-phase AC power, typically at a voltage level of 110 kV, is introduced from the urban power grid into the main substation. Through the main transformer, the voltage is stepped down to medium-voltage three-phase AC power at levels such as 35 kV or 10 kV. The reduced-voltage AC power is then transmitted via the medium-voltage ring network to individual traction substations. Within each traction substation, transformers and power converters further convert the electrical energy into DC power, which is finally supplied to trains for traction operation.

The primary function of the traction power supply system is to provide sufficient and stable electrical energy to electric locomotives or electric multiple units (EMUs), enabling the generation of traction force for train operation while ensuring efficient energy utilization. Urban rail transit systems typically adopt high-power traction configurations to support rapid train startup, acceleration, and high-speed operation. At present, traction power levels can reach several hundred kilowatts or even the megawatt range. Under such conditions, the use of a single high-power converter in a DC traction substation becomes impractical due to limitations in equipment volume, weight, and cost. As a result, modular configurations based on the parallel connection of multiple low-power converter units are being increasingly adopted in modern traction substations.

### 2.2. Configuration of the Traction Power Supply System

#### 2.2.1. AC Power Supply System

**Figure 2:**
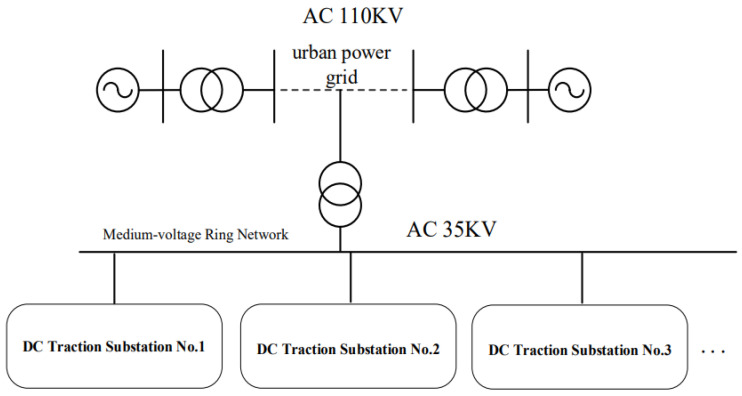
Structure of the AC power supply system.

The AC power supply system serves as the primary source of electrical energy for the entire traction power supply system. It mainly includes the urban power grid, the main substation, the medium-voltage ring network, and downstream traction substations. Electrical energy generated by power plants is transmitted to the urban power grid through step-up transformers and high-voltage transmission lines. The voltage level of the urban power grid is used as the incoming supply for the main substation of the traction power supply system. Through the main substation transformer, the voltage is stepped down and delivered to DC traction substations, as illustrated in Figure 2.

In typical urban rail transit applications, high-voltage three-phase AC power is drawn from the urban power grid at a voltage level of approximately 110 kV. Depending on specific application scenarios, centralized, distributed, or hybrid power supply schemes may be adopted. In these schemes, the 110 kV three-phase AC power is stepped down to medium-voltage levels, such as 35 kV, and transmitted to traction substations within the supply section via medium-voltage cables. This configuration ensures reliable power delivery while maintaining operational flexibility and redundancy within the traction power supply network.

#### 2.2.2. DC Traction Power Supply System

**Figure 3:**
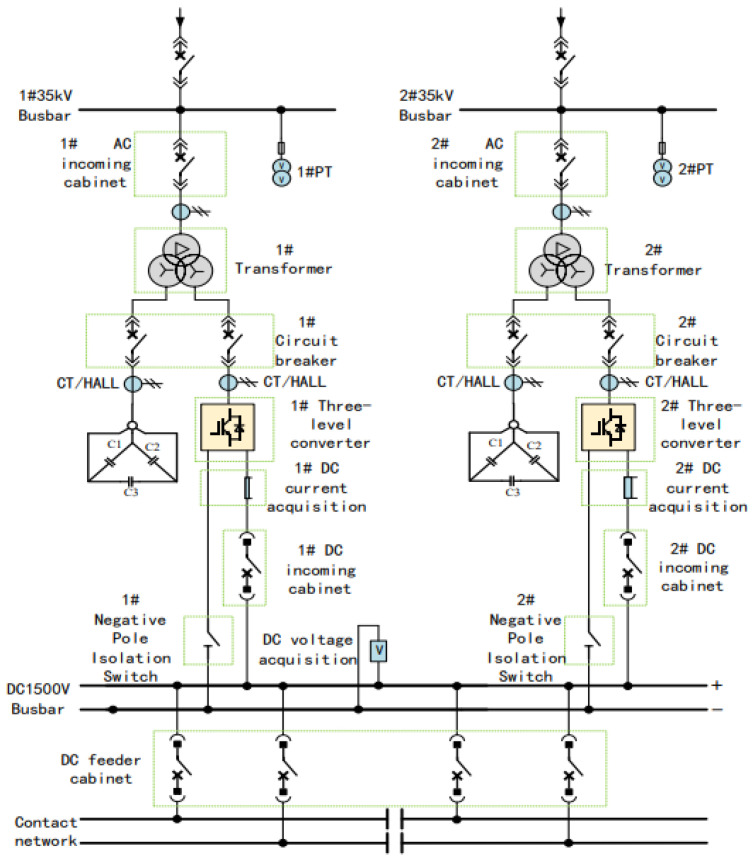
Main circuit of the DC traction power supply system.

The DC traction power supply system mainly consists of traction transformers, three-level converters, AC incoming cabinets, and DC incoming cabinets. Its primary functions include power conversion, power distribution, and fault protection. The main wiring configuration of the DC traction power supply system is shown in Figure 3.

Within the traction substation, the traction transformer is used to step down the medium-voltage three-phase AC power supplied by the medium-voltage ring network to low-voltage three-phase AC power suitable for converter operation. In the system considered in this study, the transformer reduces the voltage from 35 kV to 0.9 kV. The three-level converter then converts the low-voltage three-phase AC power into DC power at a voltage level of 1500 V, which meets the operating requirements of urban rail transit vehicles. In addition to supplying traction power, the three-level converter can also operate in inverter mode to convert regenerative braking energy from DC to AC and feed it back into the power grid, thereby replacing conventional regenerative energy feedback devices.

The AC incoming cabinet and DC incoming cabinet are responsible for system-level protection and isolation. When abnormal operating conditions such as short circuits or overloads occur, these cabinets can rapidly disconnect the faulty sections to protect the traction power supply system and associated electrical equipment. Through coordinated operation with the traction transformer and the power converter, the DC traction power supply system ensures safe, reliable, and flexible power delivery under both traction and braking conditions.

The three-level converter adopted in this paper enables the integrated realization of rectification and inversion functions, effectively replacing the traditional combination of a diode rectifier and an independent regenerative energy feedback converter. This integrated configuration supports four-quadrant operation of urban rail transit systems and significantly reduces equipment redundancy. The topology employed is the Active Neutral Point Clamped (ANPC) three-level converter, in which fully controlled semiconductor devices are used as neutral-point clamping elements. By controlling the switching states of different clamping devices, multiple redundant zero states can be generated, allowing optimization of internal circulating current paths.

**Figure 4:**
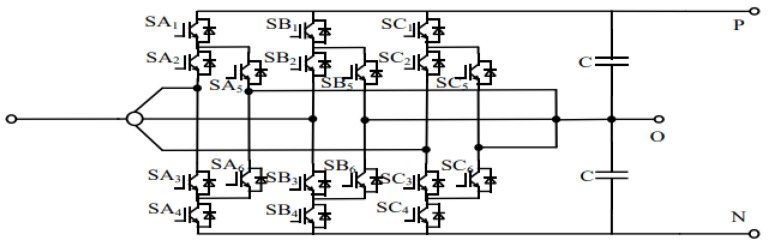
Topology of the three-level ANPC converter.

On the DC-side of the ANPC three-level converter, three output terminals—denoted as P, O, and N—are provided. The corresponding topology is illustrated in Figure 4 [20].

Compared with other three-level converter topologies, the use of redundant zero states in the ANPC structure helps to mitigate uneven loss distribution among switching devices and improves overall system efficiency. Moreover, each switching device is subjected to only half of the DC bus voltage, which reduces device voltage stress and relaxes component selection requirements. As a result, the ANPC three-level topology is well suited for flexible DC traction power supply networks characterized by low voltage and high current operation, as well as wide operating ranges and high short-circuit tolerance requirements [22,23].

#### 2.2.3. Traction Network Power Supply System

The traction network power supply system provides the direct electrical connection between traction substations and rail transit vehicles. It is mainly composed of DC feeder lines, running rails (welded ground), return conductors, and the catenary system. These components together form the traction power supply network, which is responsible for delivering DC power to trains and completing the traction current return path [24].

(1) DC feeder line

The DC feeder line is responsible for establishing the current loop between the traction substation and the catenary. Its primary function is to transmit the standardized DC voltage required by the train from the traction substation to the catenary, ensuring stable and reliable power supply during traction and braking operation.

(2) Running rails (welded ground)

The running rails support train operation mechanically and also serves as conductors for traction current return. Since the rails are conductive and insulated from the ground except at specific locations, they carry a significant portion of the traction return current together with the ground.

In the simulation model, the resistance and inductance of the positive rail are Rc = 7.8 mΩ/km and Lc = 2.66 mH/km, respectively. The resistance and inductance of the negative rail are Rr = 20 mΩ/km and Lr = 0.78 mH/km. In addition, the rail-to-ground resistance is set to Rg = 30 Ω/km. These parameters are determined based on the technical requirements for 60 kg/m steel rails (the mainstream rail type for urban metro in China) specified in the national standard Code for Design of Metro (GB 50157-2013) [7] and are consistent with the actual measured data of urban rail transit traction networks and classic research results in this field [16,24]. These values are highly consistent with the on-site measured parameters of the real traction network of a Tianjin metro line (the engineering background of this study), tested under the condition of a 25 °C normal operation without rail joint faults. The actual measured values are Rc = 7.78 mΩ/km, Lc = 2.52 mH/km, Rr = 20.3 mΩ/km and Lr = 0.771 mH/km (the relative deviation from the simulation settings is less than 5%). The rail-to-ground resistance of the real network is statistically distributed in the range of 28~32 Ω/km for the concrete sleeper with insulated rail fastening track bed, and 30 Ω/km is the typical measured value.

Rail inductance is composed of internal inductance (caused by the magnetic field generated by current flowing inside the steel rail conductor) and external inductance (caused by the magnetic field in the air outside the rail). For the 60 kg/m steel rail adopted in this study, the internal inductance accounts for approximately 15–20% of the total rail inductance (the internal inductance of the positive rail is about 0.40–0.53 mH/km, and that of the negative rail is about 0.12–0.16 mH/km), which exerts three key effects on the flexible DC traction power supply system: (1) It increases the total inductance of the traction network loop, thus reducing the rise rate of DC-side short-circuit fault current and providing a critical time margin (2–3 ms) for the activation of the proposed bypass thyristor + adaptive crowbar protection scheme; (2) It reduces the resonant frequency of the LC resonant loop formed by the traction network inductance and DC-link capacitors during PO/ON bipolar short-circuit faults, and weakens the amplitude of capacitor voltage oscillation to suppress DC capacitor overvoltage; (3) It causes a slight voltage drop along the rail with the change in traction current, and the protection threshold of the di/dt + ΔI protection criterion for the DC feeder cabinet is corrected according to this characteristic to improve the accuracy of fault identification and avoid protection maloperation.

The rail longitudinal resistance and inductance change under the large current is mainly caused by the skin effect (for resistance) and magnetic saturation of steel rail (for inductance). For the 60 kg/m steel rail (the mainstream type in urban rail transit) adopted in this study, the short-circuit fault current of the system is effectively suppressed by the proposed protection scheme (fault peak current suppression rate >70%). Even for the most severe PN bipolar short-circuit fault (the maximum total fault peak current is about 75 kA), the large cross-sectional area of the continuously welded steel rail and the low magnetic permeability of the rail material result in a very limited degree of skin effect and magnetic saturation: the variation rate of the rail longitudinal resistance is less than 5%, and the variation rate of the longitudinal inductance is less than 8%. Moreover, this variation is a slow dynamic process, which is far slower than the ultra-fast transient rise process of the fault current (up to 10 kA/ms) in the three-level converter system, and will not affect the transient characteristics of the fault current in the key time window (2–3 ms) of protection action.

The core research of this paper focuses on the DC-side fault characteristics of the three-level ANPC converter and the design/verification of the protection scheme, and the rail longitudinal resistance and inductance are the peripheral parameters of the system. In the PSCAD simulation modeling and hardware-in-the-loop (HIL) experiment of this study, we have set a ±10% parameter tolerance for the traction network parameters (including rail parameters) in advance, which fully covers the actual variation amplitude of rail resistance and inductance (5%/8%). In addition, the original study has verified that the error between the simulation and experimental results is less than 5%, and this minor variation in rail parameters will not change the core conclusions of the fault characteristic analysis and protection scheme performance verification.

**Figure 5:**
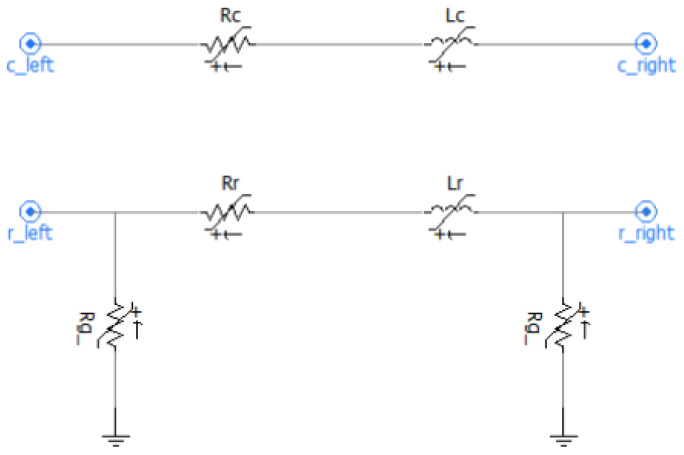
Equivalent model of the traction network and rails.

Here, R_c_ and L_c_ represent the electrical parameters of the positive rail, R_r_ and L_r_ correspond to those of the negative rail, and R_g_ denotes the rail-to-ground resistance. The equivalent simulation structure of the rail model is shown in Figure 5.

(3) Return Power Supply Line

The return conductor is used to transmit traction current from electric locomotives or electric multiple units back to the traction substation, thereby completing the electrical return path of the traction power supply system.

(4) Catenary system

The catenary system is installed along and above the rail line, allowing trains to draw electrical energy through a pantograph or current collector. At present, catenary systems used in DC traction power supply networks can be classified into overhead catenary systems and third-rail systems.

In general, overhead catenary systems are adopted for traction power supply networks with a voltage level of 1500 V, while third-rail systems are more commonly used for 750 V networks. However, with ongoing technological development, third-rail systems have also been increasingly applied in traction power supply networks operating at 1500 V.

### 2.3. Simulation Modeling and Parameter Settings

The main circuit of the DC traction substation in the traction power supply system of a subway project in Tianjin is modeled and simulated using the PSCAD/EMTDC platform. In the DC traction power supply system considered in this study, the rated power of the traction load is 3.75 MW, while the short-term maximum power reaches 7.5 MW. These parameters are determined in accordance with the traction power matching requirements for urban rail transit EMUs specified in the national standard Code for Design of Metro (GB 50157-2013) [7], and are consistent with the actual operational parameters of the Tianjin metro project (the engineering background of this study) as well as the industry-recognized traction substation capacity configuration principles [2,6,16]. Under such high-power operating conditions, the application of a single high-power converter is impractical due to limitations related to equipment volume, weight, and cost. Therefore, a modular parallel configuration consisting of multiple low-power converter units is adopted in this project.

**Figure 6:**
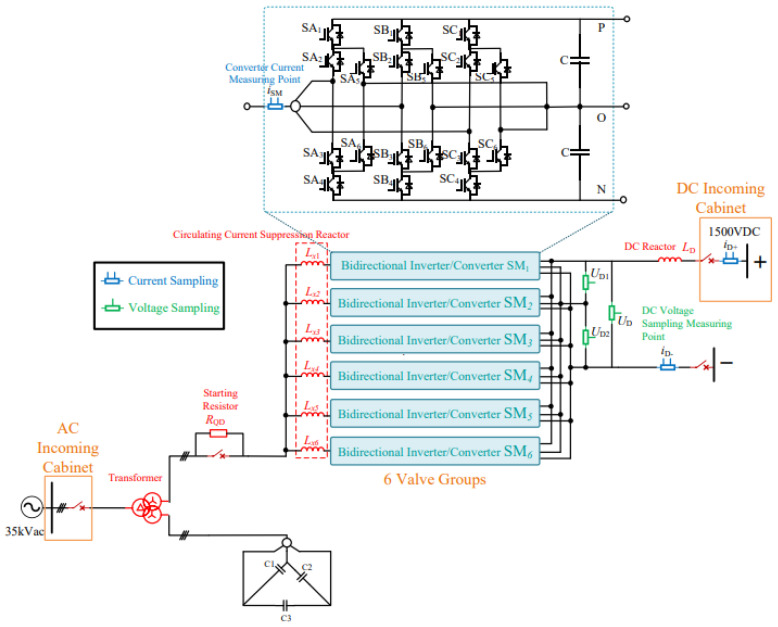
Main circuit of the DC traction substation.

Considering both sensor cost and system complexity, the simulation model is developed with a minimal number of voltage and current measurement points while still ensuring accurate fault detection and transient analysis. The main circuit of the DC traction substation, together with the key voltage and current sampling locations required for subsequent fault analysis, is shown in Figure 6.

On the AC side, a three-phase voltage source with a voltage level of 35 kV is used to represent the medium-voltage ring network of the urban power grid. The traction transformer is modeled as a conventional three-winding transformer. The selection of the transformer equivalent reactance is mainly determined by voltage harmonic distortion, current harmonic performance, and short-circuit current levels. At the same time, the transformer turns ratio is designed to improve DC voltage utilization while avoiding excessively low modulation indices of the converter.

The power converter adopts a modular parallel configuration consisting of six ANPC three-level converter units. On the AC side, the converters are connected in parallel for each phase, while on the DC-side, the P, O, and N terminals of all converter units are connected correspondingly. Although this configuration offers a simple and practical implementation, it may introduce zero-sequence circulating currents among parallel converters. To suppress such circulating currents, an additional inductor L_x_ is introduced on the AC side of each converter module [25,26,27].

On the DC-side, both the upper and lower DC capacitors are composed of six parallel-connected capacitors with a capacitance of 1700 μF each. After determining the DC capacitor configuration, a starting resistor is incorporated to control the capacitor charging process and ensure that the charging time satisfies system requirements. Special attention is paid to limiting the peak current and peak power of the starting resistor to acceptable levels, thereby ensuring safe and reliable system startup.

Based on the above modeling configuration, the main circuit parameters of the DC traction substation are determined according to engineering requirements and typical operating conditions of urban rail transit systems. The selected parameters are intended to ensure realistic representation of system behavior, accurate reproduction of fault transients, and reliable evaluation of protection performance.

The traction power supply system adopts a DC voltage level of 1500 V, which is widely used in urban rail transit applications. On the AC side, the three-phase power supply voltage is set to 35 kV to represent the medium-voltage ring network. The traction transformer is configured with a turns ratio of 35:0.9:0.9, matching the voltage requirements of the three-level converter and ensuring sufficient DC voltage utilization.

To control the initial charging process of the DC capacitors and limit inrush current during system startup, a starting resistor with a resistance value of 0.1 Ω is introduced. The DC-side filter capacitor is set to 300 μF, while the DC smoothing reactor is modeled with an inductance of 0.2 μH to suppress current ripple and improve DC-side dynamic performance.

On the AC side, an inductance of 13 μH is added to suppress circulating currents among parallel converter modules. The power semiconductor devices used in the three-level converter are modeled based on the Infineon FF1800R17IP5 IGBT/diode module, whose electrical characteristics and current-carrying capability are suitable for high-current traction applications.

For the DC energy storage unit, the upper and lower DC capacitors are each composed of six parallel-connected capacitors with a capacitance of 1700 μF, forming a symmetrical DC-link structure. The switching frequency of the converter is set to 1950 Hz, taking into account the trade-off between switching losses, harmonic performance, and control bandwidth.

Performance comparison of several protection schemes is shown in Table 1.

The simulation model is established using the PSCAD/EMTDC platform, with key parameters justified based on typical metro project specifications (GB 50157-2013 Code for Design of Metro [7]) and engineering practice. The detailed main circuit parameters of the DC traction substation are summarized in Table 2.

**Figure 7:**
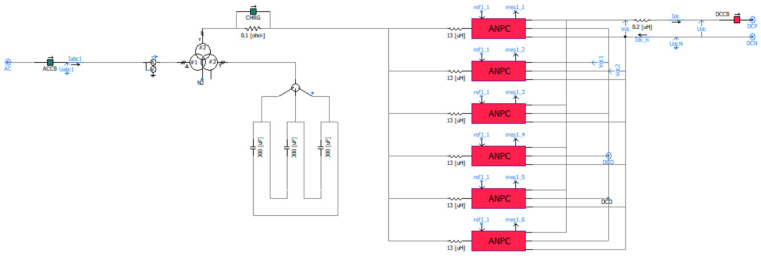
PSCAD/EMTDC simulation model of the flexible DC traction power supply system.

Based on the established system configuration and parameter settings, a detailed PSCAD/EMTDC simulation model of the flexible DC traction power supply system is developed. The distances to adjacent traction substations are set to 3097.5 m and 3250 m, which are typical spacing values for urban metro traction substations (3–4 km). Adjacent traction substations are modeled as DC voltage sources to simulate fault current feedback. The system can switch between traction and braking modes, and supports bidirectional power flow as shown in Figure 7.

**Figure 8:**
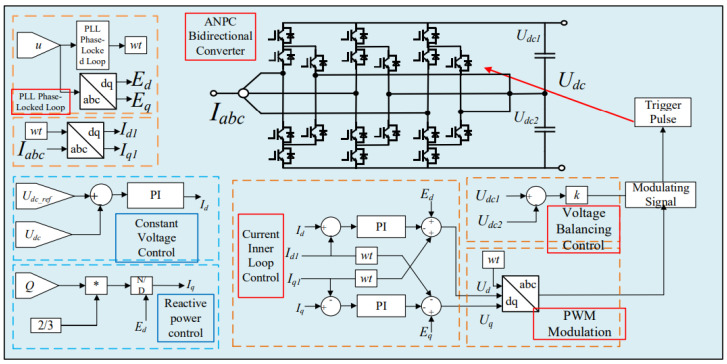
Control structure of the bidirectional three-level ANPC converter.

As can be seen from Figure 7, the system comprises key components including an AC power supply, a traction transformer, a three-level ANPC converter, DC-link capacitors and a traction network. This model fully reflects the energy flow paths under both traction and braking operating conditions, providing an accurate system foundation for the subsequent analysis of short-circuit fault mechanisms and the design of protection strategies. The operating process and control principle of the converter are illustrated in Figure 8 [31,32,33,34].

**Figure 9:**
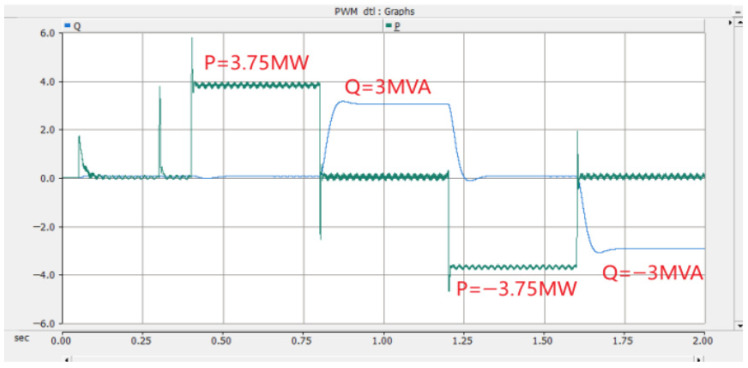
Simulation waveforms under traction operating conditions.

**Figure 10:**
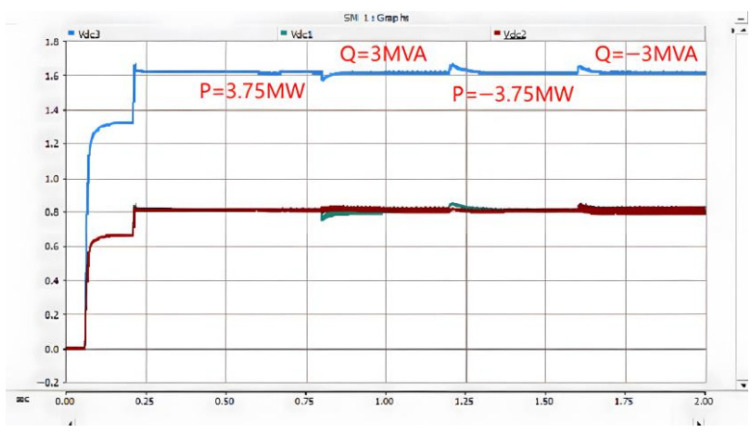
Simulation waveforms under regenerative braking conditions.

Under the double-loop control, the locomotive can achieve four-quadrant operation of active and reactive power, and the operating voltage of the locomotive can be controlled according to the set voltage reference value. The simulation is carried out under the simulated traction power supply system with the following conditions: the AC switch is blocked at 0.06 s and the starting resistor starts charging; the isolating switch is closed at 0.16 s and the starting resistor stops charging; the converter pulses are unlocked and the converter starts operating at 0.21 s; power is input at 0.4 s and the locomotive starts working. Taking the four-quadrant power operation as an example, the simulation waveforms are shown in Figure 9 and Figure 10.

Figure 9 shows the waveform diagram of the locomotive’s operating power. The locomotive operates in the traction mode with a power of 3.75 MW from 0.4 s to 0.8 s, in the inductive reactive power mode with 3 MVA from 0.8 s to 1.2 s, in the regenerative feedback mode with a power of 3.75 MW from 1.2 s to 1.6 s, and in the capacitive reactive power mode with 3 MVA from 1.6 s to 2 s. It can be seen from the simulation results that the above-mentioned control strategy enables the four-quadrant operation of the locomotive.

Figure 10 shows the voltage waveforms at the DC-side output of the bidirectional converter, where Vdc3 denotes the total output voltage at the DC terminal, and Vdc1 and Vdc2 represent the voltages of the upper and lower DC-link capacitors of the three-level bidirectional converter, respectively. It can be seen from the simulation results that the bidirectional converter can well achieve voltage balancing of the upper and lower capacitors, and the total voltage can be stabilized around the reference value of 1.6 kV, thus ensuring the stable operation of the locomotive.

### 2.4. Protection Functions of DC Traction Power Supply System

Short-circuit faults may generate enormous short-circuit currents that cause damage to electrical equipment such as transformers and converters. Therefore, it is crucial to implement necessary protection measures against such short-circuit faults. By configuring the switching operations of the AC incoming line cabinet on the AC side, as well as the DC incoming line cabinet and DC feeder cabinet on the DC-side in accordance with fault characteristics, most faults including equipment overload, overcurrent and overvoltage can be effectively protected against. This reduces the impact of short-circuit faults on equipment, thereby protecting the equipment or extending its service life. This section mainly elaborates on the protection actions and protection logic of the AC and DC incoming line cabinets.

#### 2.4.1. AC Incoming Line Cabinet

**Figure 11:**
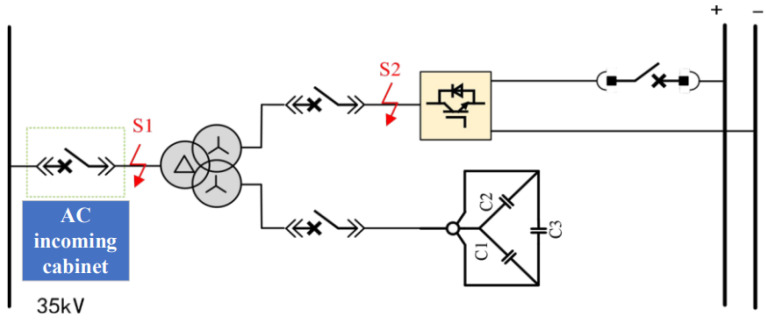
Location of short-circuit faults on the AC side of traction power supply system.

The AC incoming line cabinet is used to connect the external AC power supply to the internal AC busbar of the traction power supply system, and is also equipped with a relay protection device inside. When faults such as three-phase short circuit, two-phase-to-ground short circuit, overload, and cable single-phase-to-ground short circuit occur in the AC-side system of the traction substation, the relay protection device acts in accordance with the protection settings to trip the circuit breaker and protect the electrical equipment. Location of short-circuit faults on the AC side of traction power supply system is shown in Figure 11.

In DC traction power supply systems, the common protection methods for AC switchgear cabinets include the processes detailed below.

##### High-Current Tripping Protection

High-current tripping protection is the intrinsic protection of circuit breakers. It can quickly identify fault information and execute action commands with an extremely short time delay, generally ranging from 20 to 40 ms, and serves as the main protection of the protection device. Due to its fast operation response, and in order to reduce the number of its maloperations, the setting value is generally set relatively large, resulting in a narrow protection range. To accurately identify fault currents, the setting value can be set to be slightly higher than 300% of the rated load current (i.e., the maximum load current):

(1) $$I_{hc}=K_{hc}\times I_{N}$$

where *I_N_* is the rated current of the AC system, and *K_h__c_* is the protection safety factor, which is generally set to be greater than 3.

##### Overcurrent Protection

As the backup protection for the main protection, overcurrent protection initiates protective actions with a certain time delay when the main protection fails or the fault current does not reach the setting value of the main protection. In accordance with the sensitivity requirements of relay protection, the time delay of overcurrent protection is generally set between 0.2 and 0.35 s in microcomputer-based protection devices, and its setting value is lower than that of the tripping protection:

(2) $$I_{oc}=K_{oc}\times I_{N},T_{oc}=0.2–0.35\;s$$

where *K_oc_* is the protection safety factor, with a value ranging from 2.5 to 3.

##### Overload Protection

Overload protection is mainly designed to provide protection for the power supply system in the event of an overload fault. When the system operates under an overload condition, the overload protection is activated and issues an alarm signal after a certain time delay. The setting value of overload protection is generally not higher than the current value at the maximum load power, and the time delay is relatively long, typically more than 10 s.

Therefore, its setting criteria are as follows:

(3) $$I_{ol}=K_{ol}\times I_{M},T_{ol}=10–30\;s$$

##### Zero-Sequence Overcurrent Protection

Zero-sequence overcurrent protection is used to detect and isolate single-phase and two-phase ground short-circuit faults in AC cables. The three-phase load of the system is balanced during normal operation, with no zero-sequence current generated. When a single-phase or two-phase ground short-circuit fault occurs in the system, the symmetry of the three-phase system is broken and zero-sequence current is induced in the system. Its setting criteria are as follows:

(4) $$I_{lx}=K_{lx}\times I_{N},T_{ol}=0.2–0.4$$

where *K_lx_* is the safety factor, with a value ranging from 0.2 to 0.4.

#### 2.4.2. DC Incoming Line Cabinet

**Figure 12:**
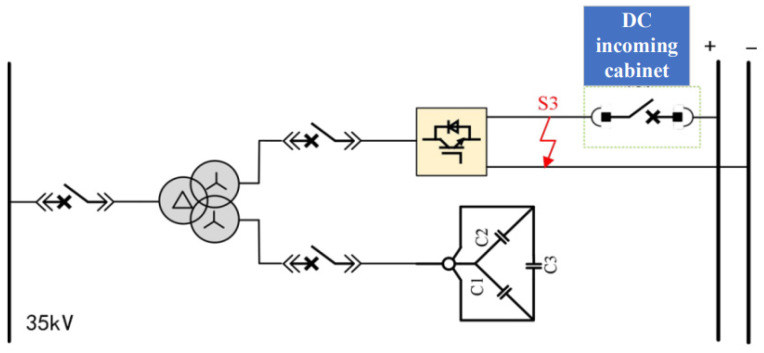
Location of short-circuit faults on the DC-side of traction power supply system.

When a bipolar short-circuit fault occurs on the DC-side busbar of the traction power supply system, phenomena such as a sudden current surge and undervoltage will occur. The DC incoming line cabinet is used to protect against such faults, and is generally equipped with high-current tripping protection, reverse current protection, overcurrent protection and other functions. Since the converter in the system analyzed in this paper is a bidirectional converter, the DC switchgear cabinet is not equipped with reverse current protection. Location of short-circuit faults on the DC side of traction power supply system is shown in Figure 12.

##### High-Current Tripping Protection

High-current tripping protection serves as the main protection in the DC incoming line cabinet. It can quickly diagnose faults and initiate protective actions with a time delay of approximately 10 ms. When a short-circuit fault occurs on the busbar of the traction power supply system, adjacent traction substations inject current into the fault point, resulting in a high peak value of the short-circuit current and thus a narrow protection range for this protection scheme. To accurately identify the fault current, its protection setting value is defined as follows:

(5) $$I_{gz}=K_{gz}\times I_{dc\_m,}\;T_{gz}=10\;ms$$

where *I_dc___m_* is the DC current value of the system operating at the maximum power and minimum voltage, and *K_g__z_* is the safety factor, generally set to 1.5 to 2.

##### Overcurrent Protection

As the backup protection for the main protection, overcurrent protection initiates protective actions with a certain time delay when the main protection malfunctions or the fault current fails to reach the setting value of the main protection. Its setting principle satisfies the following formula:

(6) $$I_{gzs}=K_{gzs}\times I_{dc\_m},\;T_{gzs}>T_{gz}$$

where *K_gz_* is the reliability coefficient, with a value ranging from 1.3 to 1.5.

#### 2.4.3. DC Feeder Cabinet

The DC feeder cabinet is used to accurately identify fault information on the DC traction network, quickly clear short-circuit faults, and maintain the safe and reliable operation of the system. It is generally equipped with *di*/*dt* + Δ*I* protection, undervoltage protection, automatic reclosing and other protection functions, among which *di*/*dt* + Δ*I* protection serves as the main protection. The *di*/*dt* + Δ*I* protection consists of the rate of current change (*di*/*dt*) protection and the current increment (ΔI) protection, and the two protection functions cooperate with each other. Specifically, the *di*/*dt* protection is mainly used to clear non-metallic short-circuit faults in the middle and remote sections, while the Δ*I* protection is mainly used to clear non-metallic faults in the middle and near sections.

The DC-side current fluctuates correspondingly when the rail locomotive starts, accelerates, decelerates and brakes. The setting value of the *di*/*dt* protection shall be selected according to the maximum rate of current change in the locomotive under the above operating conditions. The starting value of the di/dt protection is generally 1.2 to 1.5 times the maximum rate of current change during the normal operation of the locomotive, denoted as *di*/*dt_del*, and the return value of the protection is generally 0.7 to 0.8 times the maximum rate of current change in the locomotive, denoted as *di*/*dt_m*. The time delay is related to parameters such as the system voltage level and load current; in systems with a relatively high voltage level, the time delay is generally 0.2 to 0.4 s.

#### 2.4.4. Traction Network

When a fault occurs on the traction network or the locomotive operating condition changes, there will be a corresponding change in current increment. By comparing the amplitude of the current change, the Δ*I* protection can distinguish between load current and fault current. The setting value of the current increment protection is calibrated based on the maximum current increment during locomotive overload. Therefore, the maximum current of the locomotive under short-time operating conditions is generally selected for calibration:

(7) $$\Delta I_{d}=K_{d}\times I_{dc\_m}$$

where *K_d_* is the reliability coefficient, generally set to 2.0.

## 3. Analysis of DC-Side Bipolar Short-Circuit Faults in Three-Level Converters

When a DC-side bipolar short-circuit fault occurs in an ANPC three-level bidirectional converter, all power semiconductor devices are blocked immediately, and the converter enters a fault protection state. However, even after the blocking of insulated gate bipolar transistors (IGBTs), fault currents can continue to flow through the freewheeling diodes of the converter modules. Under such conditions, the freewheeling diodes are subjected to extremely high short-circuit currents, which may exceed their rated current limits and result in severe thermal stress or permanent damage. Therefore, a detailed analysis of DC-side bipolar short-circuit faults and corresponding protection strategies is essential for ensuring the safe operation of three-level converter-based traction power supply systems [28].

In urban rail transit traction power supply networks, traction substations are electrically interconnected through the catenary and running rails. As a result, when a DC-side short-circuit fault occurs at one traction substation, adjacent substations may also contribute fault current to the short-circuit point. Compared with conventional two-level converter systems, the P–O–N three-terminal DC topology of three-level converters introduces additional bipolar short-circuit fault types, including PN, PO, and ON short circuits. These fault types exhibit distinct current paths, capacitor discharge behaviors, and voltage stress characteristics, making their transient responses significantly different from those of traditional DC short-circuit faults [35].

In this study, the DC-side bipolar short-circuit behavior of the three-level ANPC converter is investigated under typical operating conditions of urban rail transit systems. The traction substation under study is located between two adjacent substations, with distances of 3097.5 m and 3250 m, respectively. During fault analysis, the AC-side network from the high-voltage side of the traction substation to the converter AC terminals is equivalently represented by an AC voltage source, while the DC-side contributions from adjacent traction substations are modeled using DC voltage sources. The system operates under steady-state load conditions before fault occurrence, and the short-circuit fault is introduced during normal operation to analyze the resulting transient characteristics [36].

Based on the above modeling assumptions, the transient current paths, capacitor voltage variations, and fault current magnitudes associated with PN, PO, and ON bipolar short-circuit faults are analyzed in detail in the following subsections [37].

**Figure 13:**
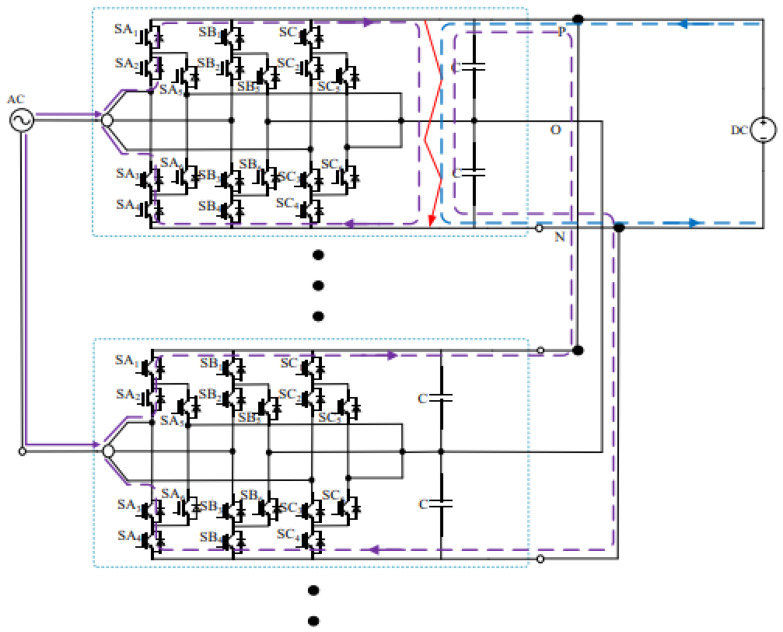
Current conduction paths of the PN bipolar short-circuit fault.

**Figure 14:**
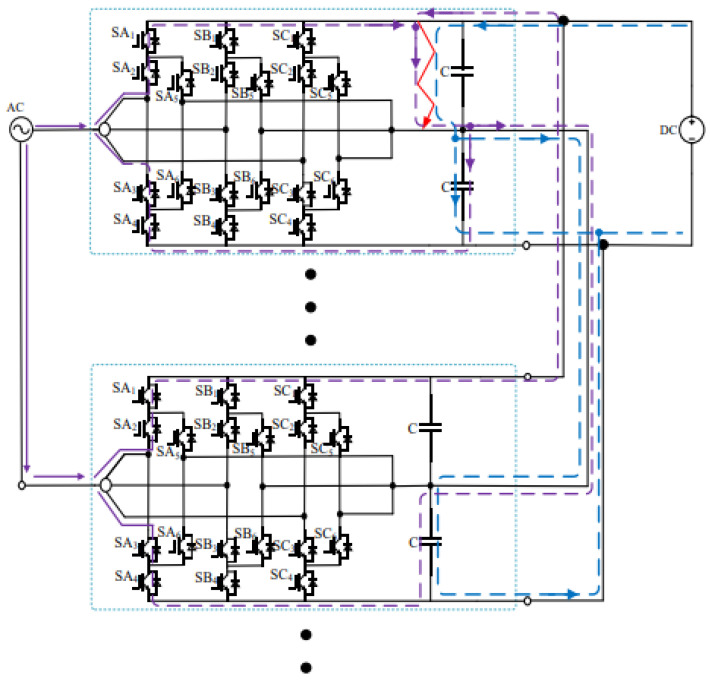
Current loop of the PO pole-to-pole short-circuit fault.

**Figure 15:**
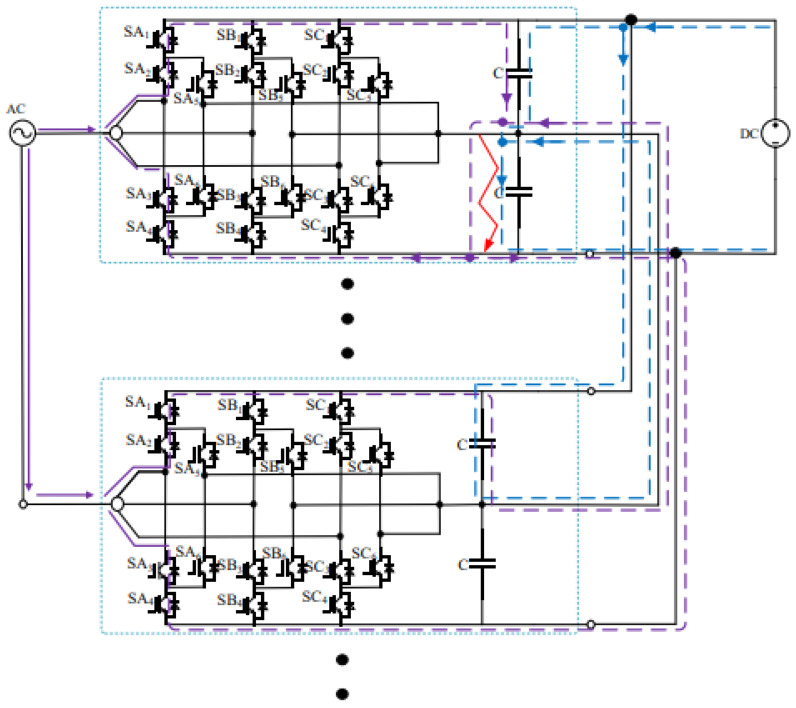
Current and voltage conduction paths of the ON bipolar short-circuit fault.

Figure 13, Figure 14 and Figure 15 show the fault current flow diagrams, in which the solid red arrows represent the main fault current paths (e.g., the capacitor discharge current and AC grid injection current in the case of PN short circuit); the dashed purple arrows denote the feedback current from adjacent substations caused by the fault at the local substation, and the dashed blue arrows indicate the discharge current from the DC capacitors of each converter to the short-circuit point at the moment of short circuit.

### 3.1. Analysis of PN Bipolar Short-Circuit Fault

Under normal operating conditions, the traction load is connected to the DC traction power supply system and operates steadily. In the simulation, the locomotive starts loaded operation at 0.4 s. At 0.6 s, a PN pole-to-pole (bipolar) short-circuit fault occurs on the DC-side of the three-level bidirectional converter. After the fault occurs, both the AC grid and the adjacent traction substations on the DC-side inject current into the short-circuit point, resulting in a rapid increase in the fault current. The current conduction paths after the fault are shown in Figure 13.

The three-level converter adopts a modular parallel configuration; therefore, the DC capacitor voltages of all converters are identical, and the AC-side currents of each converter are equal and all flow toward the fault point. As a result, the short-circuit current is shared among the parallel converter modules. The peak current conducted by the freewheeling diode in a single-phase bridge arm of each converter reaches 4.32 kA.

At the instant of the short circuit, the DC capacitors of each converter discharge rapidly into the fault point, causing a sharp decrease in the DC capacitor voltage. The discharge duration is approximately 1.2 ms, and the total discharge current of the upper and lower DC capacitors of each converter is about 13.6 kA, which can be calculated as Equation (8):

(8) $$I_{cap}=2\times C\times \frac{∆u}{∆t}=2\times 0.0102\times \frac{800}{0.0012}=13.6\;kA$$

**Figure 16:**
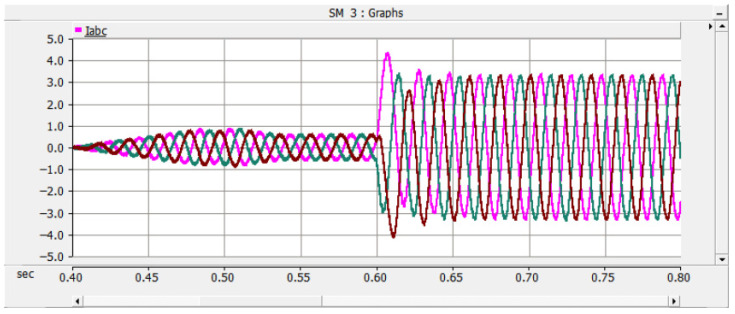
Current waveform of the converter’s AC side.

**Figure 17:**
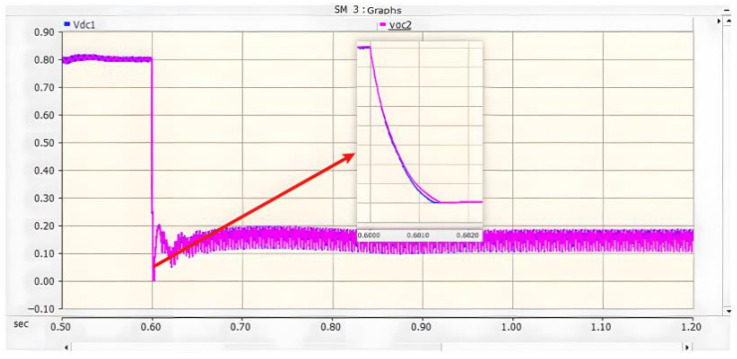
DC capacitor voltage waveform.

**Figure 18:**
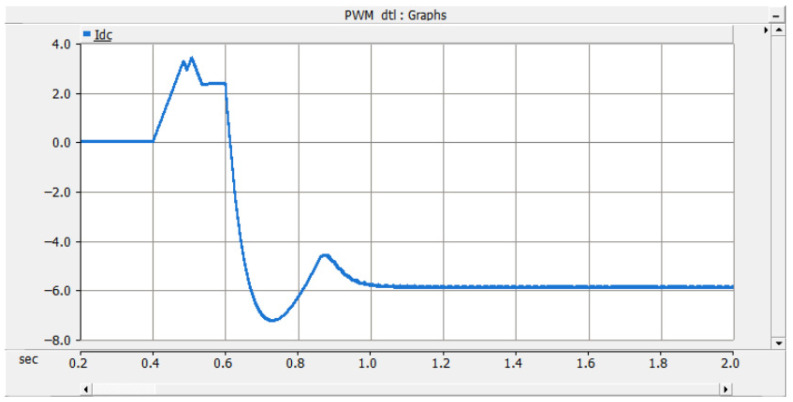
DC current waveform.

Meanwhile, the adjacent traction substations also feed current into the short-circuit point of the faulted converter through the DC traction network. The corresponding AC-side current, DC capacitor voltage, and DC current waveforms are shown in Figure 16, Figure 17 and Figure 18 [30].

In summary, the short-circuit current at the fault point $I_{gz}$ can be expressed as the superposition of the converter AC-side current, the DC capacitor discharge current, and the DC-side current contribution, which is given by Equation (9):

(9) $$I_{gz}=6I_{SM}+6I_{cap}+I_{dc}$$

where $I_{SM}$ is the sum of the upper-arm currents of a single converter, $I_{cap}$ is the DC-side capacitor discharge current, and $I_{dc}$ is the DC-side current.

Accordingly, the current flowing into the P terminal of the faulted DC bus can be expressed as Equation (10):

(10) $$5I_{cap}+I_{dc}=\;75\;kA$$

As shown in Figure 13, after the PN pole-to-pole short-circuit fault occurs, the IGBTs in the converter modules are blocked. In this case, the freewheeling diodes conduct almost the entire short-circuit current, reaching 3–4 kA, which significantly exceeds their rated current of 1800 A. If the freewheeling diodes operate under such conditions for an extended period, serious overheating and even device failure may occur.

### 3.2. Analysis of PO Bipolar Short-Circuit Fault

The locomotive starts loaded operation at 0.4 s. At 0.6 s, a PO pole-to-pole short-circuit fault occurs on the DC-side of the three-level bidirectional converter. After the fault occurs, the upper DC capacitor of the converter discharges and its voltage decreases, while the voltage of the lower DC capacitor rises. Meanwhile, both the AC grid and the adjacent traction substations on the DC-side inject current into the short-circuit point, resulting in a sharp increase in the fault current. The post-fault current conduction paths are shown in Figure 17 [38].

After the PO pole short-circuit fault occurs, the upper DC capacitor of the bidirectional converter immediately discharges to the fault point, forming a discharge loop. Since only the upper capacitor participates in the discharge process, the discharge current is half of that under the PN pole-to-pole short-circuit fault, i.e., 6.8 kA, as given by Equation (11).

(11) $$I_{cap}=C\times \frac{∆u}{∆t}=0.0102\times \frac{800}{0.0012}=6.8\;kA$$

Meanwhile, the adjacent traction substations can be regarded as DC voltage sources discharging toward the fault point, forming a loop consisting of the traction substation, fault point, lower capacitor, traction substation. Due to the inductance of the rails, the current flowing from the traction substations through the capacitor exhibits oscillatory behavior. As a result, the lower capacitor bears the full DC terminal voltage, and its voltage also oscillates accordingly.

**Figure 19:**
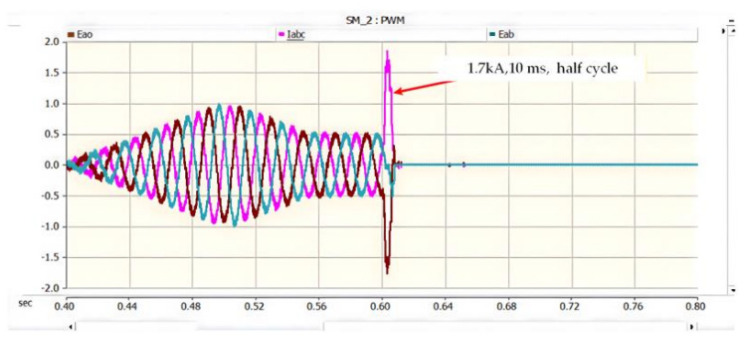
Simulation results of the PO bipolar short-circuit fault (DC-side current).

**Figure 20:**
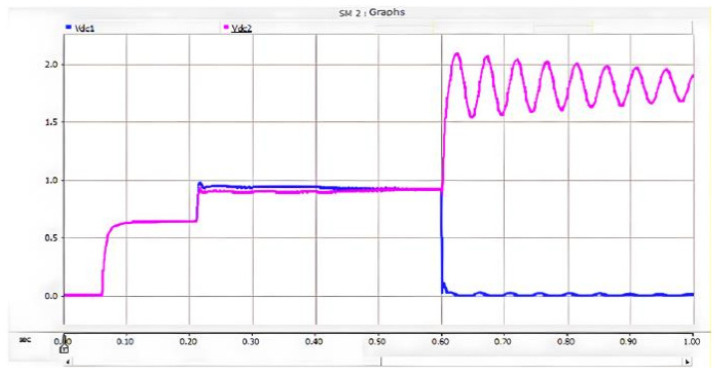
Simulation results of the PO bipolar short-circuit fault (DC capacitor voltage).

**Figure 21:**
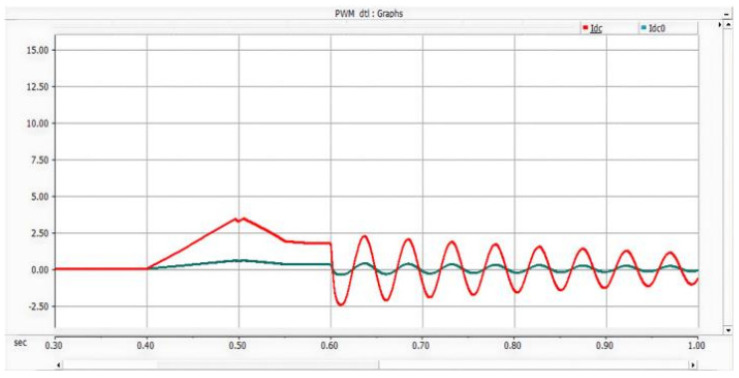
Simulation results of the PO bipolar short-circuit fault (voltage oscillation characteristics).

On the AC side of the converter, a current loop of grid–fault point–lower capacitor–grid is formed. During this process, the peak current flowing through the freewheeling diodes reaches 1.7 kA and lasts for approximately 10 ms, corresponding to half of the power-frequency cycle. When the voltage of the lower capacitor rises to the reference value, the potentials at both ends become equal, and consequently, no current flows on the AC side. The corresponding waveforms are shown in Figure 19, Figure 20 and Figure 21 [39].

In summary, the short-circuit current at the fault point $I_{gz}$ can be expressed as Equation (12)

(12) $$I_{gz}=6I_{SM}+6I_{cap}+I_{dc}$$

where $I_{SM}$ is the sum of the upper-arm currents of a single converter, which becomes zero under steady-state conditions; $I_{cap}$ is the discharge current of the upper DC capacitor of the converter; and $I_{dc}$ is the DC-side current, whose maximum value after the fault is approximately 2.5 kA.

Accordingly, the current flowing into the P terminal of the faulted DC bus can be expressed as Equation (13)

(13) $$5I_{cap}+I_{dc}=\;36\;kA$$

According to Figure 20, when a PO pole-to-pole short-circuit fault occurs in the converter, the DC terminal voltage of the faulted traction substation does not decrease because it remains connected to adjacent traction substations through the catenary and rails. Under this condition, the entire DC terminal voltage is borne by the lower DC capacitor. However, this voltage far exceeds its rated value of 1200 V, surpassing the voltage withstand capability of the DC capacitor. This may lead to dielectric breakdown and even capacitor explosion [29].

### 3.3. Analysis of ON Bipolar Short-Circuit Fault

The locomotive starts loaded operation at 0.4 s. At 0.6 s, an ON pole-to-pole short-circuit fault occurs on the DC-side of the three-level bidirectional converter. Similar to the PO pole-to-pole short-circuit fault, after the ON pole-to-pole fault occurs, the voltage of the lower DC capacitor decreases due to discharge, while the voltage of the upper DC capacitor increases. Meanwhile, both the AC grid and the adjacent traction substations on the DC-side inject current into the short-circuit point, resulting in a sharp increase in the fault current. The post-fault current conduction paths are shown in Figure 21.

After the ON pole short-circuit fault occurs, the lower DC capacitor of the bidirectional converter immediately discharges toward the fault point, forming a discharge loop. The discharge current is still half of that under the PN pole-to-pole short-circuit fault, i.e., 6.8 kA. Meanwhile, the adjacent traction substations can be regarded as DC voltage sources discharging toward the fault point, forming a loop consisting of the traction substation–upper capacitor–fault point–traction substation. For the same reason as in the PO pole-to-pole short-circuit fault, the upper capacitor bears the full DC terminal voltage and exhibits oscillatory behavior.

**Figure 22:**
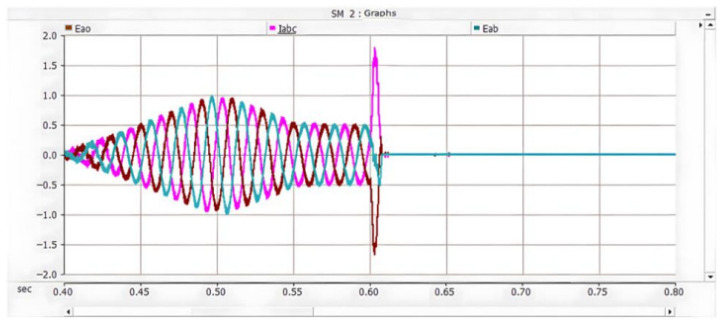
Simulation results of the ON bipolar short-circuit fault (DC-side current).

**Figure 23:**
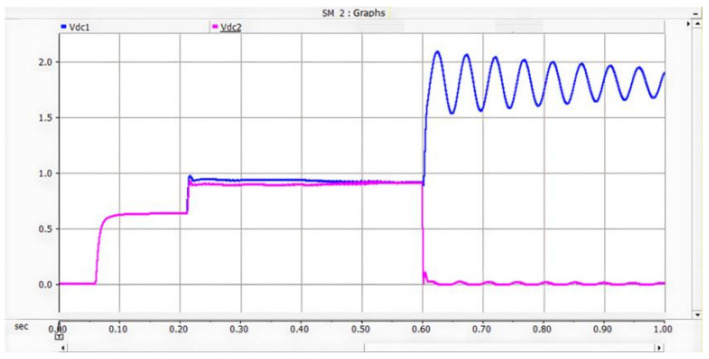
Simulation results of the ON bipolar short-circuit fault (DC capacitor voltage).

**Figure 24:**
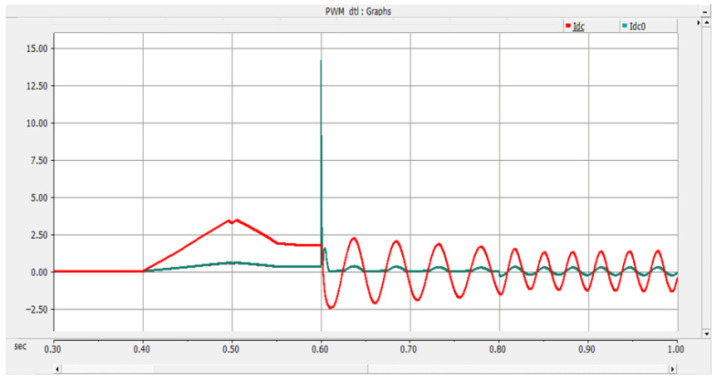
Simulation results of the ON bipolar short-circuit fault (voltage redistribution).

On the AC side of the converter, a current loop of grid–upper capacitor–fault point–grid is formed. During this process, the peak current flowing through the freewheeling diodes reaches 1.7 kA and lasts for approximately 10 ms, corresponding to half of the power-frequency cycle. When the voltage of the lower capacitor rises to the reference value, the potentials at both ends become equal, and consequently, no current flows on the AC side. The corresponding waveforms are shown in Figure 22, Figure 23 and Figure 24.

In summary, the short-circuit current at the fault point $I_{gz}$ can be expressed as Equation (14):

(14) $$I_{gz}=6I_{SM}+6I_{cap}+I_{dc}$$

where $I_{SM}$ is the sum of the upper-arm currents of a single converter, which becomes zero under steady-state conditions; $I_{cap}$ is the discharge current of the DC-side capacitor of the converter; and $I_{dc}$ is the DC-side current, whose maximum value after the fault is approximately 2.5 kA.

When an ON pole-to-pole short-circuit fault occurs at the DC terminal of the converter, the resulting fault risk is almost equivalent to that of the PO pole-to-pole short-circuit fault. The difference is that a PO pole-to-pole short-circuit fault may damage the lower DC capacitor of the converter, whereas an ON pole-to-pole short-circuit fault may lead to damage of the upper DC capacitor.

## 4. Protection Scheme Design and Simulation Analysis for DC-Side Bipolar Short-Circuit Faults

### 4.1. Analysis of Protection Schemes for DC-Side Pole-to-Pole Short-Circuit Faults of the Converter

When a PN pole-to-pole short-circuit fault occurs on the DC-side of the converter, the peak current on the secondary side of the transformer reaches 20 kA, and the IGBTs are blocked. The current flowing through the freewheeling diode of a single converter reaches 3.3 kA, which poses a serious overcurrent risk. Therefore, it is necessary to introduce bypass thyristors to transfer the short-circuit current and protect the converter.

When a PO or ON pole-to-pole short-circuit fault occurs on the DC-side of the converter, the current from adjacent traction substations flows into the lower capacitor or the upper capacitor, respectively. In this case, the entire DC terminal voltage is borne by the lower or upper capacitor of the converter. Due to the inductance of the rails, the voltage oscillates at approximately 1.6 kV, resulting in an overvoltage risk for the DC capacitors. Therefore, a crowbar circuit needs to be triggered to bypass the short-circuit current and keep the capacitor voltage within a safe range [29,38,39].

In this study, three cases are analyzed for the PN pole-to-pole short-circuit fault: installing a fast fuse on the P line of the DC-side of the faulted converter, installing a fast fuse on the N line of the DC-side of the faulted converter, and installing fast fuses on both the P and N lines of the DC-side of the faulted converter.

When a fast fuse is installed on the P line of the DC-side of the faulted converter, the positive half-cycle of the AC-side fault current flows entirely through the upper-arm diodes of the faulted converter, passes through the short-circuit point, and is then evenly distributed to the lower arms of the six converters. In this case, the current borne by the upper-arm diodes of the faulted valve group reaches 20 kA. After the fast fuse blows, the DC-side fault current flows through the upper capacitors of the healthy converters, along the neutral line, and through the upper-arm diodes of the faulted converter to form a loop with the short-circuit point. This further increases the current stress on the upper-arm diodes, and the upper capacitors of the healthy converters are also exposed to an overvoltage risk.

When a fast fuse is installed on the N line of the DC-side of the faulted converter, the positive half-cycle of the AC-side fault current is evenly distributed among the upper-arm diodes of the six converters, passes through the short-circuit point, and then flows entirely through the lower-arm diodes of the faulted converter. In this case, the current borne by the upper-arm diodes of the faulted valve group reaches 20 kA. After the fast fuse blows, the DC-side fault current flows from the short-circuit point through the lower capacitor of the faulted converter and is then evenly distributed via the neutral line to the lower capacitors of the other five healthy converters. As a result, the lower capacitor of the faulted converter experiences overvoltage, and the lower capacitors of the healthy converters are also subject to overvoltage risk.

When fast fuses are installed on both the P and N lines of the DC-side of the faulted converter, the AC-side fault current flows entirely through the faulted converter, and the freewheeling diodes of the faulted converter bear the entire AC-side short-circuit current. After the fast fuses blow, the DC-side fault current flows through the capacitors of the healthy converters, causing overcurrent risks for both the upper and lower capacitors of the healthy converters.

Based on the above analysis, it can be concluded that when a PN pole-to-pole short-circuit fault occurs on the DC-side of the converter, installing fast fuses on the DC P bus, the DC N bus, or both the DC P and N buses cannot effectively protect the freewheeling diodes and DC capacitors of the converter. On the contrary, such measures may even lead to further damage to the circuit.

**Figure 25:**
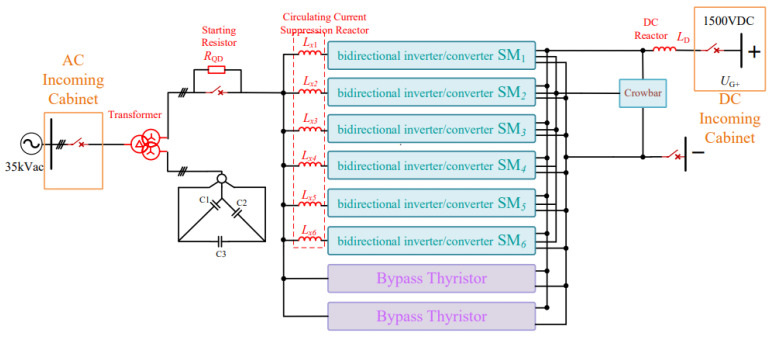
Main circuit of the proposed protection scheme based on bypass thyristor and crowbar circuit.

Therefore, a combined protection scheme based on bypass thyristors and a crowbar circuit is adopted in this paper to protect the converter against DC-side pole-to-pole short-circuit faults. The main circuit of the traction substation after adding the bypass thyristors and crowbar circuit is shown in Figure 25 [40,41,42].

### 4.2. Simulation Experiments

To verify the feasibility of the bypass thyristor and crowbar protection scheme proposed in the previous sections, simulations are carried out in PSCAD for three types of DC-side pole-to-pole short-circuit faults, namely PN, PO, and ON faults, with the bypass thyristor and crowbar circuits incorporated.

During the short-circuit simulations, the short-circuit resistance is set to $R_{f}=10\;m\Omega$. The distance between two adjacent traction substations is 3 km. The DC voltage reference is $U_{dc\_ref}=1.6\;\mathrm{kV}$. The locomotive operates at the rated power of P=3.75 MW. The startup time is $t_{0}=0.4\;s$, and the short-circuit fault occurs at $t_{s}=0.6\;s$.

**Figure 26:**
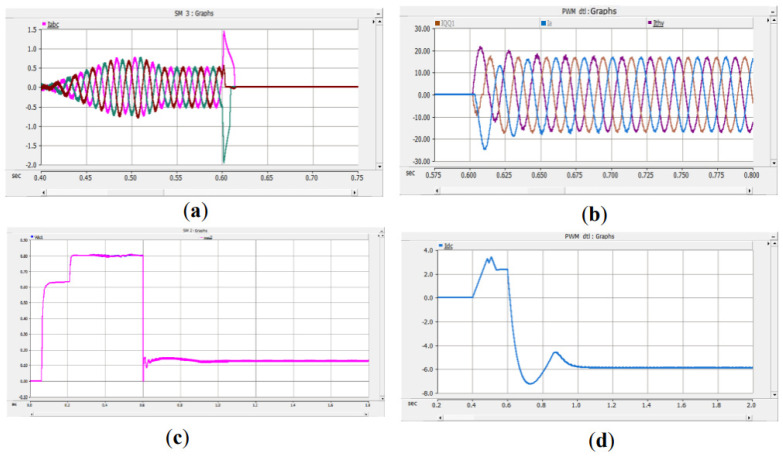
PN bipolar short-circuit protection verification: (**a**) AC-side current of the converter; (**b**) current of the bypass thyristor; (**c**) DC-link capacitor voltage; (**d**) DC-side current.

As shown in Figure 26, after the PN pole-to-pole short-circuit fault occurs, the AC-side current of the converter rises sharply, and the DC bus voltage instantaneously drops to zero. When the current of any phase on the transformer AC side exceeds the DC bus short-circuit overcurrent protection threshold $I_{set1}$, the DC bus short-circuit protection is triggered, and the firing criterion of the bypass thyristor is activated. The bypass thyristor is able to turn on rapidly and divert the short-circuit current that would otherwise flow through the converter. The simulation results indicate that the AC-side current of the converter lasts only about 10 ms with a peak value of approximately 2 kA, which satisfies the repetitive peak current requirement of 3.6 kA for the diodes in the FF1800R17P5 module.

**Figure 27:**
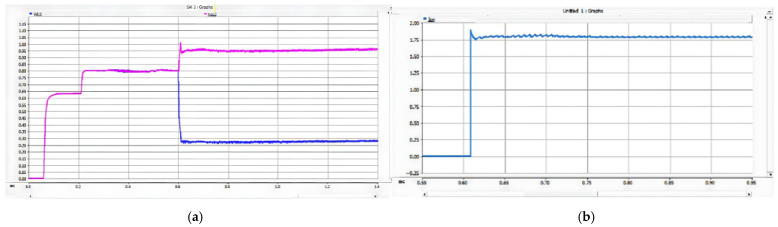
PO bipolar short-circuit protection verification: (**a**) DC-link capacitor voltage waveform; (**b**) crowbar current waveform.

**Figure 28:**
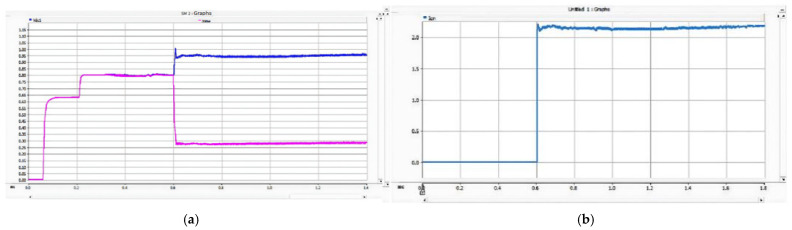
Simulation verification of the ON bipolar short-circuit protection scheme: (**a**) DC-link capacitor voltage waveform; (**b**) crowbar current waveform.

As shown in Figure 27 and Figure 28, when a PO or ON pole-to-pole short-circuit fault occurs, the voltage of the lower or upper DC capacitor rises rapidly. When the capacitor voltage exceeds the crowbar activation threshold, the DC bus overvoltage protection is triggered, and the IGBT in the crowbar circuit is turned on immediately. The simulation results show that the voltage of the lower or upper capacitor is stabilized at approximately 1.1 kV, which satisfies the rated voltage requirement of 1200 V for the MLC-LL capacitor.

These results demonstrate that the proposed bypass thyristor and crowbar protection scheme can protect the three-level bidirectional converter effectively and accurately against PN pole-to-pole short-circuit faults as well as PO and ON pole-to-pole short-circuit faults. The simulation results verify that the proposed protection scheme exhibits excellent reliability and fast response characteristics, thereby compensating for the lack of effective protection research for neutral-line-related pole-to-pole short-circuit faults in three-level converter-based traction power supply systems.

### 4.3. Bypass Thyristor and Crowbar Circuit Action Control Logic

**Figure 29:**
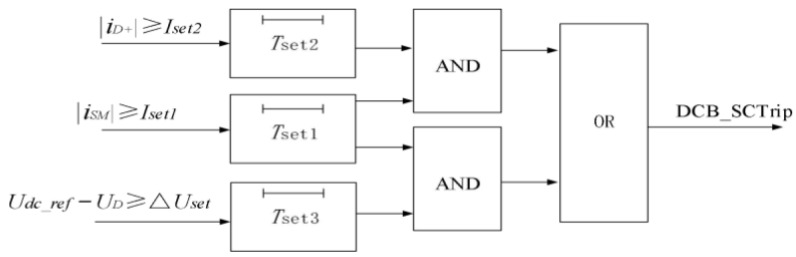
DC short-circuit protection criterion.

There are generally two causes of converter overcurrent: first, a bipolar short-circuit fault on the DC-side of the converter; second, a short-circuit fault between the DC positive bus and ground. Based on this, the DC bus short-circuit protection criteria shall simultaneously satisfy either “the DC current is greater than its current trigger value and the converter AC-side current is greater than its current trigger value” or “the converter AC-side current is greater than its current trigger value and the difference between the DC terminal voltage and the given value is greater than its trigger value”. The protection flow is shown in Figure 29.

Upon DC bus short-circuit protection, all converter IGBT pulses are blocked and the bypass thyristor is fired.

**Figure 30:**
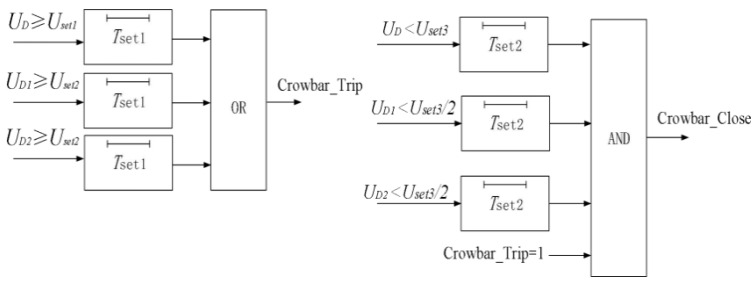
DC bus overvoltage protection flowchart.

The crowbar start-stop protection criteria are the trigger criteria for: ① short-circuit faults between the neutral line and the DC positive pole, DC negative pole, or ground; ② DC bus voltage rise caused by energy imbalance of the DC bus. The crowbar start protection is triggered when the voltage of the DC bus or either the upper or lower capacitor of the converter exceeds its protection setting value. The crowbar bypass shall be turned off when the DC bus voltage is lower than the shutdown setting value, or when the voltage of either the upper or lower capacitor is lower than the shutdown setting value. The protection flow is shown in Figure 30.

The Crowbar_Trip signal activates full crowbar engagement; the Crowbar_Close signal removes all crowbars.

## 5. Experimental Validation and Analysis

### 5.1. Experimental Platform and Configuration

To further verify the effectiveness and engineering feasibility of the proposed protection scheme, a hardware-in-the-loop (HIL) experimental platform was established. The experimental setup was implemented based on a real-time simulation system and was used to validate the protection performance of the three-level ANPC converter under DC-side bipolar short-circuit fault conditions.

The HIL experimental platform is based on the Modeling-Tech MT 6020 real-time simulator, with the following key specifications:

Real-time simulation step: 50 μs;
Sampling rate: 20 kHz;
Delay between simulation and controller: <100 μs;
DSP controller: TMS320F28337 (32-bit floating-point DSP, clock frequency 200 MHz).

Power cabinet: Water-cooled heat dissipation system (operating temperature < 60 °C).

The platform interfaces the real-time simulation model (PSCAD) with the physical DSP controller, enabling closed-loop testing of the protection scheme under realistic fault conditions. The interaction delay between the simulation model and the physical DSP controller is <100 μs; fiber-optic transmission provides ultra-low-latency support for “multi-device cascading,” ensuring real-time performance.

**Figure 31:**
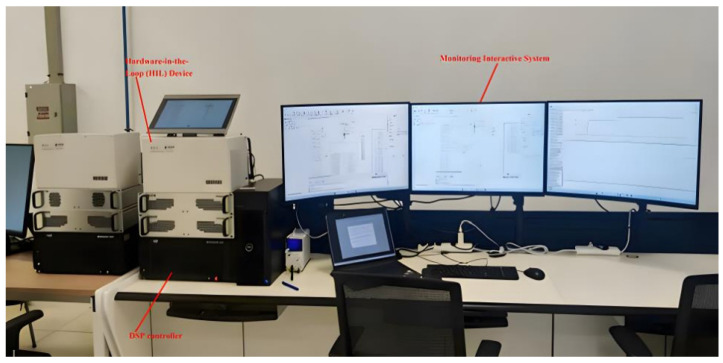
Hardware-in-the-loop (HIL) experimental platform.

The overall configuration of the HIL experimental platform is shown in Figure 31.

**Figure 32:**
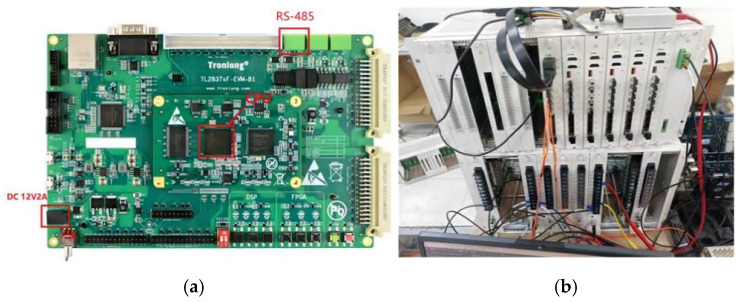
Control hardware and DSP-based controller: (**a**) DSP control board; (**b**) Modeling-Tech MT 6020 controller.

The HIL platform mainly consists of a real-time simulation device, a DSP-based control unit, as shown in Figure 32, and a human–machine interface (HMI). The real-time simulator is responsible for executing the traction power supply system model, while the DSP controller performs the protection logic and control algorithms. The HMI is used for parameter configuration, real-time monitoring, and fault event visualization.

**Figure 33:**
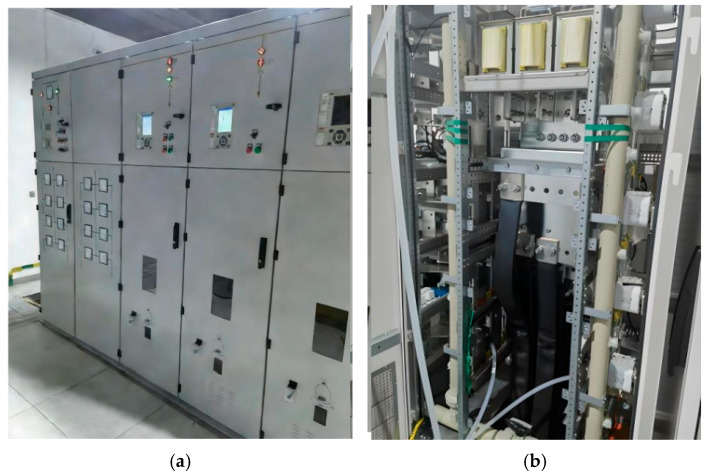
Power cabinet and cooling system of the experimental platform: (**a**) DC switchgear and DC feeder cabinet, (**b**) water-cooled heat dissipation bypass thyristor and Crowbar circuit.

In this paper, except for the bipolar short-circuit fault at the DC terminal of the three-level converter, which requires the bypass thyristor and crowbar circuit for protection in the early stage of the fault, almost all other faults are protected and cleared by the AC switchgear, DC switchgear, and DC feeder cabinet. In addition, during overload or short-circuit faults, large currents cause the system to generate heat. Therefore, a water-cooled heat dissipation design is also adopted in this project to reduce the operating temperature of the components, thereby significantly improving the service life and reliability of the circuit components, as shown in Figure 33.

### 5.2. Experimental Results Under DC-Side Bipolar Short-Circuit Faults

To further verify the protection function of the bypass thyristor + crowbar circuit proposed in this paper for the bipolar short-circuit faults at the DC terminal of the three-level converter, functional tests were conducted on a hardware-in-the-loop (HIL) platform. The tests mainly include the protection performance of the bypass thyristor + crowbar circuit when PN, PO, and ON bipolar short-circuit faults occur at the DC terminal of the three-level converter, with the fault occurrence time set to 0.8 s in the experiment.

#### 5.2.1. PN Bipolar Short-Circuit Fault Experimental Results

**Figure 34:**
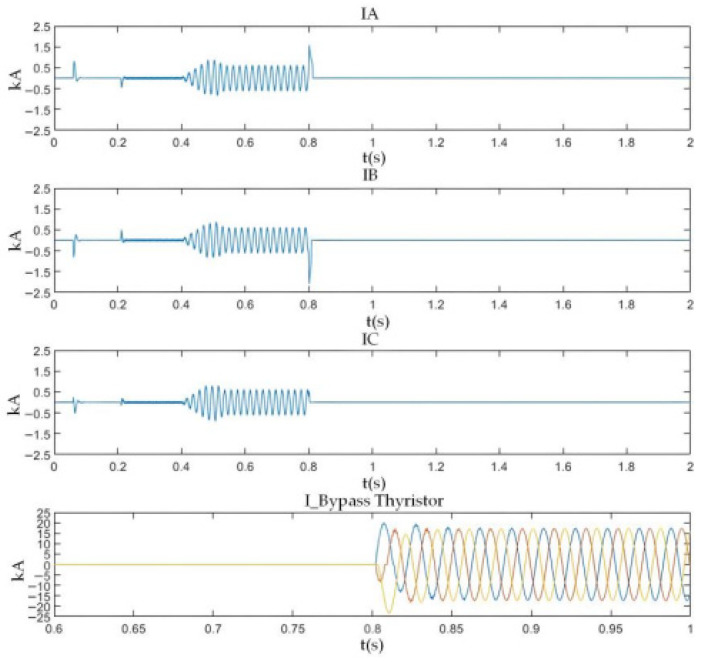
Experimental results of the PN bipolar short-circuit fault.

As can be seen from the experimental waveform in Figure 34, after the occurrence of the PN bipolar short-circuit fault, the peak values of the AC-side phase currents of the converter are measured as follows: the phase-a current reaches 1.58 kA at 0.8019 s, the phase-b current peaks at 2.08 kA (the maximum among the three phases) at 0.8019 s, and the phase-c current reaches 0.52 kA at 0.8017 s. Meanwhile, the peak current of the bypass thyristor reaches 20.12 kA.

The experimental data confirm that the bypass thyristor can rapidly divert the fault current within 2 ms after the fault occurs, effectively limiting the converter’s peak current within the withstand capacity of the freewheeling diodes. Specifically, the maximum peak AC-side current (2.08 kA for phase-b) represents an increase of approximately 15.6% compared to the rated current of 1.8 kA for the freewheeling diodes in the FF1800R17IP5 module. This peak current lasts for about 10 ms, which is well within the diodes’ repetitive peak current capability (3.6 kA), achieving a safety margin of ~42.2%. Additionally, the fault duration is significantly shortened from the natural decay time of ~50 ms to 10 ms, which effectively avoids excessive thermal accumulation and potential device degradation.

#### 5.2.2. PO Bipolar Short-Circuit Fault Experimental Results

During a PO fault, the ~1.6 kV oscillation of the lower DC-link capacitor stems from the series LC tank formed by the traction-line inductance and the DC capacitor; energy cyclically swaps between electric (capacitor) and magnetic (inductor) fields. With parameters taken from the actual project, the circuit is under-damped. At fault inception, the lower capacitor is pre-charged to 750 V (half of the 1.5 kV bus). DC energy injected by adjacent substations adds to the capacitor discharge, producing a resonant overshoot in the LC loop. Because the loop resistance is small, losses are low and the voltage peaks at 1.55 kV (≈1.6 kV), matching both simulation and experimental results.

**Figure 35:**
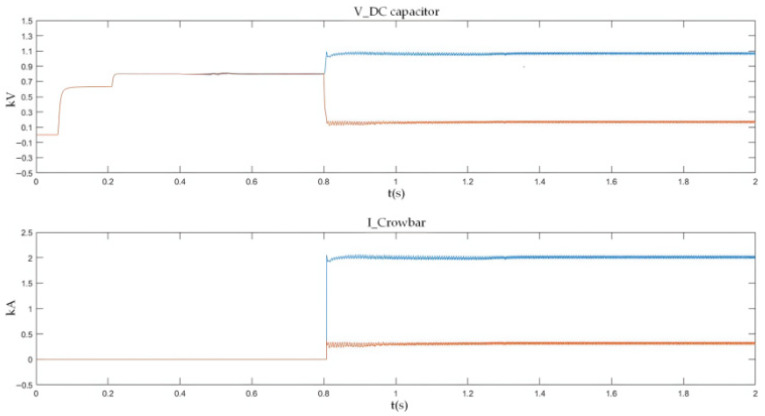
Experimental results of the PO bipolar short-circuit fault.

As shown in Figure 35, after the occurrence of the PO bipolar short-circuit fault, the voltage of the lower DC capacitor rises to 1.09 kV and finally fluctuates around 1.05 kV. The peak current flowing through the IGBT and energy-dissipating resistor in the lower bridge arm of the crowbar circuit reaches 2.05 kA. The experimental data indicate that the crowbar circuit can divert the DC current immediately after the fault occurs, thus preventing the voltage of the DC capacitor from exceeding its rated value; meanwhile, the current in the crowbar circuit does not exceed the continuous current rating of its IGBT.

The experimental results for the PO bipolar short-circuit fault are shown in Figure 35.

#### 5.2.3. ON Bipolar Short-Circuit Fault Experimental Results

**Figure 36:**
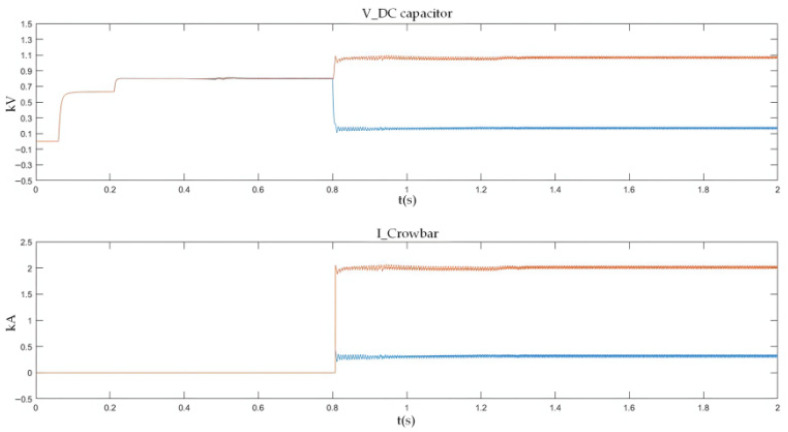
Experimental results of the ON bipolar short-circuit fault.

Figure 36 presents the experimental waveforms for the ON bipolar short-circuit fault. Similar to the PO fault case, the overvoltage stress is transferred to the opposite DC capacitor. Once the crowbar circuit is triggered, the capacitor voltage rise is effectively limited, and the system returns to a stable operating state after fault clearance.

The experimental results show that the turn-on time of the thyristor is approximately 1.6 ms (including trigger delay), and the activation time of the crowbar circuit is about 1.4 ms. The bus voltage is clamped within the range of 1.02–1.10 pu. The errors between the experimental waveforms and simulation results are less than 5% in terms of peak current, DC voltage, and capacitor voltage variation. During multiple repeated tests, no thermal runaway occurred in the bypass thyristor, the platform recovery time was around 40–50 ms, and no inrush current was observed. These results further verify the engineering feasibility of the proposed protection and energy management system.

The hardware-in-the-loop experimental results demonstrate that the protection technology based on the bypass thyristor + crowbar circuit can rapidly and accurately identify the occurrence of PN bipolar short-circuit faults as well as PO and ON bipolar short-circuit faults in the three-level bidirectional converter, and can also effectively achieve the protection function, thus verifying the engineering application value of this method.

### 5.3. Discussion on Simulation Results and Experimental Validation

#### 5.3.1. Rationality of Simulation Modeling and Interpretation of Simulation Protection Effects

The simulation model adopts a modular parallel configuration of six ANPC three-level converters, which effectively simulates the high-power operation scenario of traction substations. The consideration of adjacent traction substations as DC voltage sources and the equivalent modeling of rail inductance fully reflect the mutual coupling between traction networks, making the fault current paths and voltage oscillation characteristics more realistic. In addition, the setting of key parameters (e.g., DC-link capacitor capacity, switching frequency) balances the accuracy of fault transient reproduction and simulation efficiency.

For PN bipolar short-circuit faults, the bypass thyristor diverts fault current within 10 ms, limiting the peak AC-side current of the converter to approximately 2 kA, which meets the repetitive peak current requirement of the FF1800R17P5 module diodes. For PO/ON bipolar short-circuit faults, the crowbar circuit stabilizes the capacitor voltage at around 1.1 kV, avoiding dielectric breakdown caused by overvoltage. These results confirm that the differentiated protection strategy (bypass thyristor for PN faults, crowbar circuit for PO/ON faults) effectively addresses the diverse risks of different fault types.

The simulation also shows that the proposed scheme suppresses fault peak currents by more than 70% and limits DC bus overvoltage within ±10% of the rated voltage. This performance meets the stringent requirements of urban rail transit for power supply continuity and equipment safety under high-density operation.

#### 5.3.2. Comparison Between Simulations and Experiments

Experimental data show that the bypass thyristor’s turn-on time is approximately 1.6 ms, and the crowbar circuit’s activation time is about 1.4 ms, both meeting the rapid protection requirements. The error between experimental and simulation results is less than 5% in terms of peak current, DC voltage, and capacitor voltage variation, indicating high consistency between the simulation model and the actual system. During repeated tests, no thermal runaway or secondary impact occurred, and the system recovery time was only 40–50 ms, verifying the protection scheme’s engineering feasibility.

The high consistency between simulations and experiments (error < 5%) stems from two aspects: first, the simulation model accurately reproduces the key components and topological characteristics of the experimental platform; second, the parameter settings (e.g., short-circuit resistance, substation spacing) in the simulation are consistent with the experimental conditions. This consistency confirms that the simulation model can be used for preliminary design and optimization of protection schemes, reducing the cost and cycle of experimental verification. A unified comparison of core indicators is shown in Table 3.

## 6. Conclusions

This study systematically addresses the DC-side bipolar short-circuit fault protection problem of flexible DC traction power supply systems based on three-level ANPC converters, focusing on the newly introduced PO/ON bipolar short-circuit faults and neutral-point-related faults derived from the P–O–N topology.

By establishing a PSCAD/EMTDC simulation model and conducting HIL experimental verification, the research clarifies the limitations of traditional fast fuse protection schemes and proposes a differentiated protection strategy combining bypass thyristors and adaptive crowbar circuits. This scheme effectively solves the protection blind zone of neutral-line-related short-circuit faults, achieving rapid fault identification and protection activation within 2–3 ms, with excellent performance in fault current suppression and overvoltage control. The research fills the technical gap in the protection of three-level converter-based flexible DC traction systems and provides an engineering-feasible protection solution for 1500 VDC and similar voltage-level traction power supply projects. Additionally, field tests were conducted on actual metro lines, validating the protection scheme under real operating conditions, collecting long-term performance data for optimization and artificial intelligence. Future research will focus on coordinated protection of multi-terminal flexible DC systems, where protection performance may differ for high-impedance faults (e.g., contact resistance > 100 mΩ) or faults elsewhere in the traction network (e.g., catenary faults), as well as on fault-prediction and self-healing control technologies based on artificial intelligence algorithms. It will explore the integration of data-driven or AI-based algorithms (e.g., machine learning, deep learning) for fault prediction and health management of protection components (e.g., thyristor aging, crowbar resistor degradation), further enhancing the safety and resilience of urban rail transit power-supply systems.

## Figures and Tables

**Table 1:** Performance comparison between proposed scheme and conventional methods.

Performance	Proposed Scheme	Fast Fuses	Standard Crowbar Circuits
Fault Clearance Time	<3 ms	10–40 ms	5–8 ms
Peak Current Suppression Rate	>70%	20–40%	30–50%
DC Bus Overvoltage	±10% of rated voltage	±15–25% of rated voltage	±20–30% of rated voltage
Adaptability to Fault Types	PN/PO/ON ^1^	PN only	PN only
Device Stress Mitigation	Effective (diodes/IGBTs)	Inadequate (capacitor overvoltage)	Partial (overcurrent remains)

^1^ P-Positive, O-Zero, N-Negative, are Output level of each phase in three-level converters, especially for the neutral-point clamped (NPC) type levels.

**Table 2:** Main circuit parameters of the DC traction substation.

Item	Value	Justification
AC power supply	35 kV	Standard medium-voltage level for urban rail transit traction substations
Traction power supply mode	1500 V DC	Widely used in Chinese metro projects (e.g., Tianjin, Shanghai, Beijing) [2,6,16]
Transformer ratio	35:0.9:0.9	Matches the input voltage requirement of the ANPC converter, ensuring DC voltage utilization > 90% [2,23]
Rail-to-ground resistance (Rg)	30 Ω/km	Typical value for urban metro track beds (concrete sleeper, insulated rail fastening) [7,16,28]
Rail inductance (Lc/Lr)	2.66 mH/km/0.78 mH/km	Based on actual rail parameters (steel rail, 60 kg/m) specified in GB 50157-2013 [7,28]
Starting resistor	0.1 Ω	Limits inrush current during capacitor charging to <1.5 kA (within device withstand capacity) [16,29]
DC-link capacitor	6 × 1700 μF	Symmetrical configuration, ensuring voltage balancing between upper and lower capacitors (unbalance < 2%) [23,30]
DC-side reactor	0.2 μH	Suppresses current ripple to <10% of rated current, improving DC-side dynamic performance [24,28]
Filter capacitor	300 μF	Balances ripple suppression and dynamic response (ripple voltage < 5% of rated voltage) [24,28]
AC-side reactor	13 μH	Suppresses zero-sequence circulating currents among parallel converter modules to <5% of rated current [25,26,27]
Switching frequency	1950 Hz	Balances switching losses (IGBT switching loss <2 kW) and harmonic performance (THD < 3%) [31,32,33,34]
Converter IGBT/Diode module	FF1800R17IP5 (Infineon)	Rated current 1800 A, voltage 1700 V, suitable for high-current traction applications

**Table 3:** Unified comparison table of core indicators (based on simulation + HIL experimental data).

Core Indicators	PN Bipolar Short-Circuit Fault	PO Bipolar Short-Circuit Fault	ON Bipolar Short-Circuit Fault	Core Logic of Difference Causes
Total Fault Peak Current	75 kA (HIL Experiment)	36 kA (HIL Experiment)	36 kA (HIL Experiment)	PN fault is caused by dual-capacitor discharge + dual-loop current superposition; PO/ON faults are caused by single-capacitor discharge + single loop, resulting in halved current
DC-Side Capacitor Charging/Discharging Current	13.6 kA (parallel discharge of upper and lower capacitors)	6.8 kA (discharge of upper capacitor only)	6.8 kA (discharge of lower capacitor only)	PN fault activates the full P-N loop, while PO/ON faults only activate a single-capacitor loop
DC-Side Capacitor Voltage Variation	0~0.8 kV (stable after rapid collapse)	Lower capacitor: 0.75~1.6 kV (oscillation)	Upper capacitor: 0.75~1.6 kV (oscillation)	PN fault has no resonance conditions; PO/ON faults form an LC resonant loop
Freewheeling Diode Peak Stress	4.32 kA (140% over rated value)	1.7 kA (56% over rated value)	1.7 kA (56% over rated value)	PN fault has multiple current paths and high energy superposition; PO/ON faults have a single current path
IGBT Stress Level	Blocked state (no direct current)	Blocked state (no direct current)	Blocked state (no direct current)	IGBTs are immediately locked after fault, and stress is borne by freewheeling diodes
Fault Current Contribution from Adjacent Traction Substations	48 kA (64% of total current)	25 kA (69% of total current)	25 kA (69% of total current)	PO/ON loops have slightly higher impedance, resulting in lower proportion of self-discharge and higher proportion of external contribution

## Data Availability

The original research results presented in this study have been included in the article. For further inquiries, please contact the corresponding author.
